# Association Between Endometriosis and Prognosis of Ovarian Cancer: An Updated Meta-Analysis

**DOI:** 10.3389/fonc.2022.732322

**Published:** 2022-03-31

**Authors:** Peng Chen, Chi-Yuan Zhang

**Affiliations:** Department of Obstetrics and Gynecology, Shengjing Hospital of China Medical University, Shenyang, China

**Keywords:** endometriosis, ovarian cancer, prognosis, meta-analysis, overall survival, progression-free survival

## Abstract

**Objective:**

Increased risk of ovarian cancer (OC) among endometriosis patients has been proposed. However, the association between endometriosis and prognosis of OC remains controversial. This study evaluated whether endometriosis had influence on the survival outcomes of OC through a meta-analysis.

**Methods:**

Relevant studies were retrieved from PubMed, Embase, and Web of Science databases and were evaluated using the Newcastle-Ottawa Quality Assessment Scale. Effect size was presented as hazard ratio (HR) and 95% confidence interval (CI). Heterogeneity test evaluation was performed using Cochran’s Q test and I^2^ statistics. Publication bias was determined using Egger’s test. Statistical analysis was performed using Stata 12.0 software.

**Results:**

Twenty-one studies involving 38641 patients were included. For the total OC, there were significant differences in overall survival (OS) [HR (95% CI)=0.67 (0.55, 0.80), P<0.001] and progression-free survival (PFS) [HR (95% CI)=0.58 (0.42, 0.81), P=0.001] between endometriosis-associated ovarian cancer (EAOC) and non-EAOC patients in the random-effects models (P<0.05). For ovarian clear cell cancer, there were significant differences in terms of OS [HR (95% CI)=0.63 (0.48, 0.83), P=0.001] and PFS [HR (95% CI)=0.67 (0.52, 0.87), P=0.002] between EAOC and non-EAOC patients in the fixed-effects models (P>0.05). Subgroup analysis suggested no significant differences between EAOC and non-EAOC in OS and PFS in the univariate analysis per subgroup, and PFS in the American subgroup (P>0.05).

**Conclusion:**

EAOC patients tended to have better OS and PFS than non-EAOC patients. Conducting higher quality prospective cohort studies with large sample sizes is recommended to confirm the authenticity of the current study’s results.

**Systematic Review Registration:**

https://inplasy.com/inplasy-2022-3-0109/.

## Introduction

Endometriosis is a type of estrogen-dependent chronic inflammatory disease and is a common gynecological condition affecting 5-10% of reproductive-aged women in the world. It is defined as the ectopic growth of endometrial glands and stroma, causing dysmenorrhea, pelvic pain and infertility (1). Although endometriosis is benign lesion, it exhibits malignant biological behaviors similar to cancer, such as local invasion, metastasis, invasion, and easy recurrence (2), and malignant transformation of endometriosis has been proposed as early as 1925 (3). Under the influence of multiple factors, ectopic ovarian endometrial cells with malignant potential gradually change their normal ectopic endometrial cystic epithelium to atypical ectopic endometrial epithelium and invasive carcinoma, which is then called endometriosis associated ovarian cancer (EAOC). Endometriosis may be a precursor lesion to specific subtypes of OC, and prevalence studies show that ovarian clear cell cancer (OCCC) and endometrioid ovarian cancer (EOC) are predominate in women with endometriosis (3–6).

Studies have shown that patients with endometriosis are at higher risk of developing ovarian cancer (OC) (7). Endometriosis is reported to be a risk factor for epithelial OC, which is associated with a 50% increase in epithelial OC risk (8). A meta-analysis of 13 case-control studies suggested that patients with endometriosis had a significantly elevated risk of specific histological subtypes of OC, including OCCC, EOC and low-grade serous OC (9). OC is the seventh most common cancer in women (10) and the 2nd gynecologic cause of cancer deaths in women worldwide (11), with a 5-year survival rate of 47.4%. Prognosis for OC patients is directly associated with tumor stage at the time of diagnosis, ranging from approximately 90% in stage I-tumors to 25% in metastatic tumors (10).

Several studies based on meta-analyses have revealed an association between endometriosis and prognosis of OC in 2014 to 2015, but inconsistent conclusions were found among them (12–14). In recent years, additional studies have been reported the differences of the prognosis of OC patients with or without endometriosis. For example, a retrospective nationwide cohort study of 32,419 women indicated that OC patients with endometriosis had longer overall survival than those without endometriosis, even after adjusting for confounding factors (15). Li et al. indicated that patients with EAOC had longer overall survival (109.8 months) than those with non-EAOC (47.4 months) (16). However, no associations between endometriosis and prognosis of OC were found in the study of Ju et al. (17). Therefore, to obtain a more comprehensive and objective result, we performed a meta-analysis to uncover the differences in terms of progression-free survival (PFS) and overall survival (OS) within EAOC and non-EAOC patients.

## Methods

### Search Strategy

According to a pre-established retrieval strategy, the relevant studies were systematically retrieved from PubMed, Embase, Web of Science databases with the retrieval time up to May 11, 2021 and without language restrictions. The search terms contained three categories: research object (“ovarian neoplasm”, “ovarian cancer”, “ovary neoplasm”, “ovary cancer”, “ovarian carcinoma”, “ovary carcinoma”), exposure factors (“endometriosis”, “endometrioses”) and outcomes (“mortality”, “survival”, “prognosis”). Two search terms in the same category are combined with “OR”, while “AND” was used between two search terms of different categories. The detailed retrieval strategies for different databases are listed in **Table S1**. Additionally, manual retrieval was carried out for the paper version of the relevant studies, and the references of the relevant reviews and included studies were also retrieved.

### Inclusion and Exclusion Criteria

Studies were included in this meta-analysis if they met the following criteria: (1) patients who were pathologically and histologically diagnosed as epithelial ovarian cancer were included; (2) PFS or OS of OC patients with or without endometriosis were reported; (3) they were retrospective or prospective cohort studies or nested case-control studies; and (4) the crude or multivariable adjusted hazard ratio (HR) and 95% confidence interval (CI) for PFS and OS were reported. Studies were excluded from this analysis if they were: (1) non-original articles, such as reviews, conference abstracts and comments; (2) the studies that provide only a figure but not a detailed HR (95% CI) to show the results of survival analysis; (3) duplicate studies or multiple studies involving the same data, with only the one with the most complete information included. On the basis of the above selection criteria, study retrieval was carried out by two independent investigators.

### Data Extraction and Quality Evaluation

Data extraction was conducted independently in accordance with the form pre-designed by two investigators. The following data were extracted, including the first author’s name, region of research, year of publication, when the study subjects were recruited, subject information, including: sample size, age, histological subtypes, adjuvant treatment, outcomes, and other confounding factors. The extracted data were exchanged and reviewed, and disagreements were settled through a thorough discussion. The methodological quality evaluation for the included studies, which involved the selection, comparability, and exposure of the included study subjects, were conducted with reference to the Newcastle-Ottawa Quality Assessment Scale (NOS) (18), which includes 8 scoring items with a full score of 9. Studies with a final score ranging from 7 to 9 are regarded as high quality, while those with a final score ranging from 4 to 6 or less than 4 are regarded as medium quality or low quality, respectively.

### Statistical Analysis

All statistical analyses were completed using Stata 12.0 software. HR and 95% CI were utilized as effect size indicators to evaluate the differences on PFS and OS of EAOC vs. non-EAOC. Cochran’s Q test and I^2^ test (19) were used to assess the heterogeneity among studies. Significant heterogeneity was determined with P<0.05 or I^2^>50%, and a random-effects model was utilized. A fixed-effects model was utilized when no significant heterogeneity was observed (P≥0.05 and I^2^ ≤ 50%). The effect of region, confounding factors adjusted or not for heterogeneity, and the pooled results were evaluated with a subgroup analysis. Publication bias evaluation was conducted using Egger’s test. If there was significant publication bias, the stability of the results of the meta-analysis was evaluated using the trim-and-fill method. The stability of the results was also evaluated using the method of elimination one by one.

## Results

### Study Retrieval

**Figure 1:**
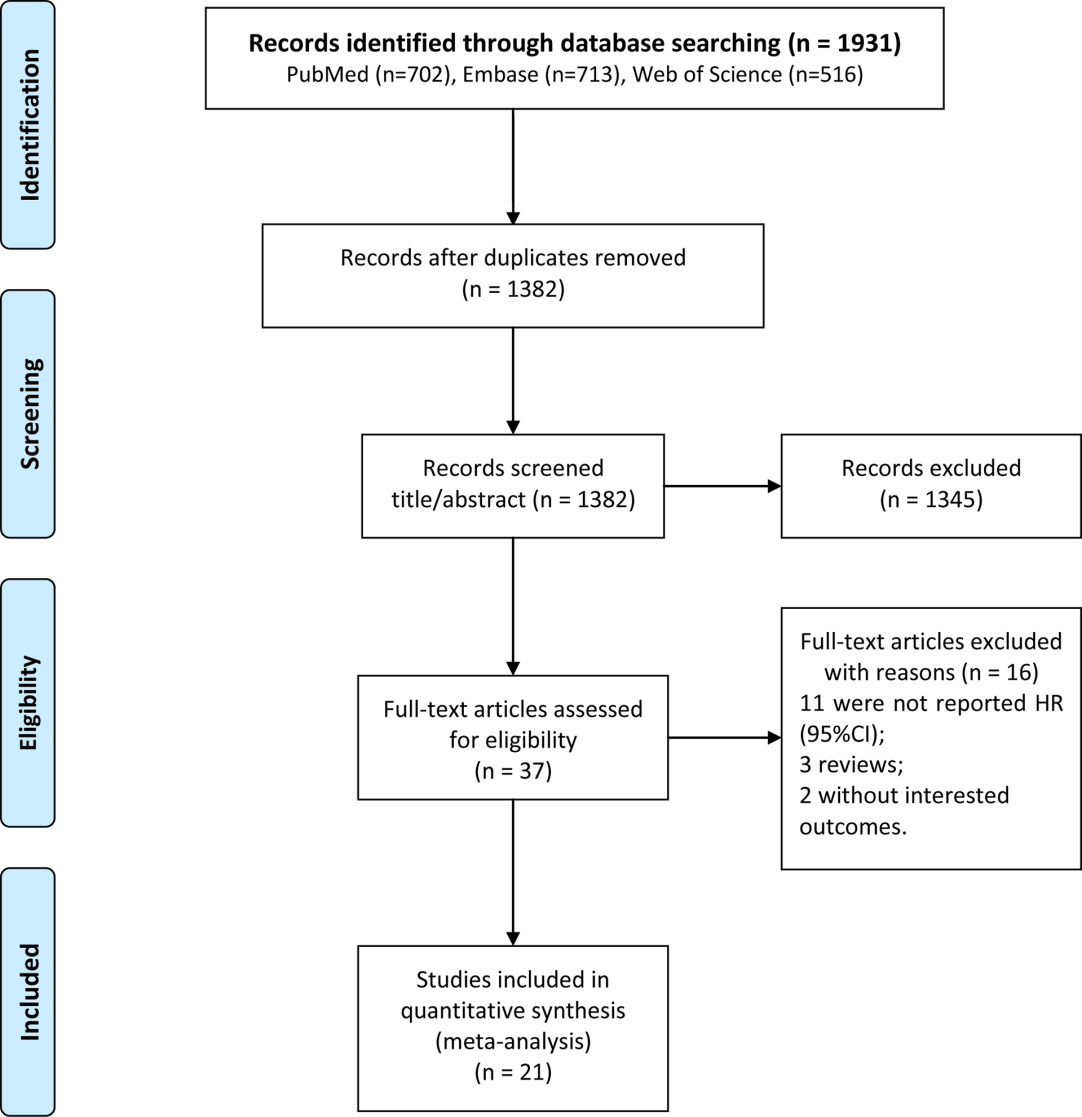
Flow chart of the patient selection process.

In total, 1931 studies, including 702 studies from PubMed, 713 studies from Embase, and 516 studies from the Web of Science database were retrieved. Among these studies, 549 duplicate studies were first removed. Of the 1382 remaining studies, 1345 irrelevant studies were excluded after title/abstract reading. After full-text reading, 16 studies were further excluded, including 11 studies without detailed HR (95% CI) to show the results, 3 reviews, and 2 studies without detailed information of PFS or OS. Finally, 21 studies (12, 15–17, 20–36) were included in the current meta-analysis (**Figure 1**).

### Characteristics of Studies

A total of 38641 cases were included in these 21 studies. **Table 1** shows the detailed characteristics of the 21 included studies. All the studies were retrospective cohort studies except for the study of Erzen et al. (23), which was a nested case-control study. The included patients had Stage I-IV OC in all studies except for the study of Ju et al. (17), in which the included patients had Stage I-III OC (data not shown). These studies were published from 1989 to 2021, and were conducted in 8 countries, such as China, South Korea, USA, Japan, and Italy. In addition, the ages of the included cases were not reported in the study of Katagiri et al. (24), but the ages of all the cases were mentioned in three studies (20, 21, 26). The remaining 17 studies reported the ages of cases in the EAOC and non-EAOC groups, and among these studies, significant differences were found between the cases belonging to different EAOC and non-EAOC age groups in ten studies (12, 15, 16, 22, 29, 31–35).

**Table 1:** Characteristics of the included studies.

Study	Area	Period of enrollment	Age, years, (EAOC/non-EAOC)	Histological subtype	n, EAOC/ non-EAOC	Adjuvant treatment	Outcomes	Adjusted factors
Barreta, A 2018 (20)	Brazil	1995-2016	56.8±11.9	OCCC, EOC	50, 40/10	NR	PFS, OS	Histology, CA-125, Stage
Crozier, MA 1989 (21)	USA	1959-1985	51 (28-77)	OCCC	59, 13/46	Radiotherapy or PBC	PFS, OS	Crude
Dinkelspiel, HE 2016 (22)	USA	2000-2013	52 (47-57)/ 61 (55-70) *	Serous, mucinous, OCCC, EOC, mixed	139, 49/90	PBC	PFS, OS	Crude
Erzen, M 2001 (23)	Slovenia	1990-1999	54.5±11.5/54.7±11.7	Serous, mucinous, OCCC, EOC, others	290, 58/232	NR	OS	Crude
Hermens, M 2021 (15)	the Netherlands	1990-2015	56 (49-63)/ 66 (56-75) *	Serous, mucinous, OCCC, EOC, others	35530, 2008/33522	Chemo- therapy	OS	Age, Surgery, Chemotherapy, stage, Histological Subtype, grade.
Ju, UC 2019 (17)	Korea	2004-2016	49.0±12.7/53.4±13.6	OCCC, EOC	119, 30/89	Chemo- therapy	PFS, OS	Stage, Grade, Laterality of tumor, LN metastasis, RD
Katagiri, A 2012 (24)	Japan	NR	NR	OCCC	60, 28/32	PBC	PFS, OS	Crude
Kim, HS 2014 (13)	Korea	1997-2012	31(<55), 16(≥55)/ 26(<55), 36(≥55) *	OCCC	109, 47/62	Taxane/PBC	PFS, OS	Age, FIGO stage, histology, optimal debulking, adjuvant treatment
Kumar, S 2011 (25)	USA	1992-2002	54/59	Serous, mucinous, OCCC, EOC	226, 42/184	NR	OS	Age, Race, Stage, Grade, treatment
Lee, HY 2020 (26)	Korea	1995-2015	51 (25-81)	OCCC	308, 107/201	PBC	PFS, OS	Age, ECOG PS, Grade, Stage, chemotherapy, Optimal debulking
Li, QW 2019 (16)	China	2002-2017	48.65±8.98/54.39±9.05 *	OCCC, EOC	128, 34/94	NR	OS	Serum CA125, RD, Ascites, Stage, Histological type, Chemo-resistance
Lu, J 2017 (27)	China	1995-2010	49.6(47.6-51.7)/51.1 (49.5-52.7)	OCCC, EOC, Mixed	196, 58/138	PBC	PFS, OS	Age, Histology, Stage, ECOG PS, LNM, RD, ascites, CA125
Noli, S 2013 (28)	Italy	1990-2010	51.4±9.8/54.3±11.2	OCCC, EOC	113, 36/77	NR	OS	Stage
Orezzoli, JP 2008 (29)	USA	1975-2002	45-61/54-63 *	OCCC	84, 41/43	PBC	OS	Age, stage, RD, chemotherapy, peritoneal washings
Paik, ES 2018 (30)	Korea	2002-2015	45.1±7.0/46.7±10.3	OCCC, EOC	224, 41/183	Taxane/PBC	PFS, OS	Age, CA-125, FIGO stage, Grade, RD
Park, JY 2018 (31)	Korea	1991-2012	48 (29-69)/51 (28-79) *	OCCC	155, 78/77	PBC	PFS, OS	CA-125, FIGO stage, Ovarian surface involvement, Optimal debulking
Ren, T 2017 (32)	China	2000-2012	45 (40-49.5)/52 (44-62) *	OCCC, EOC	304, 68/236	PBC	PFS, OS	Age, Menopause, Stage, Histotype, LNM, RD, CA125, Tumor side, Chemotherapy, Chemoresistance
Scarfone, G 2014 (33)	Italy	1990-2012	51.4±10.0/58.4±11.2 *	OCCC	73, 27/46	NR	OS	Stage
Wang, S 2013 (34)	China	2000-2012	45.8±11.2/51.2±12.7 *	EOC	188, 32/156	PBC	PFS	Age, Menopausal status, Stage, RD, Grade, Clear cell mixed, EC
Ye, S 2014 (35)	China	2000-2012	46 (30-60)/54 (30-74) *	OCCC	200, 79/131	PBC	PFS, OS	Age, CA 125, Stage, RD, Menopausal status, Gravidity
Zhu, CC 2021 (36)	China	2010-2020	45.50±6.19/50.09±10.40	OCCC	86, 16/70	Paclitaxel and platinum	PFS, OS	Crude

EAOC, Endometriosis-associated ovarian carcinoma; EOC, endometrioid ovarian carcinoma; LNM, Lymph node metastasis; OCCC, ovarian clear cell carcinoma; OS, Overall survival; PBC, Platinum-based chemotherapy; PFS, Progression-free survival; RD, Residual disease; USA, the United States of America. *means significant differences were found between the EAOC and non-EAOC groups.

### Quality Evaluation

The methodological quality evaluation for the 21 included studies was conducted with reference to NOS, and the results are shown in **Table 2**. The NOS scores of the 21 studies ranged from 5 to 8 points, of which 5 points was evaluated for one study (23), 6 points for five studies (21, 22, 24, 33, 36), 7 points for six studies (16, 20, 25, 28, 29, 31) and 8 points for nine studies (12, 15, 17, 26, 27, 30, 32, 34, 35). Overall, the methodological quality of the included studies was moderate. Recall bias and confounding bias were the primary bias.

**Table 2:** Quality assessment of the included studies.

Study	Representati- veness of the exposed cohort	Selection of the unexposed cohort	Ascertainment of exposure	Outcome of interest not present at start of study	Control for important factor or additional factor	Outcome assessment	Follow-up long enough for outcomes to occur	Adequacy of follow-up of cohorts	Total quality scores
Barreta, A 2018 (20)	☆	☆	☆	--	☆	☆	☆	☆	7
Crozier, MA 1989 (21)	☆	☆	☆	--	--	☆	☆	☆	6
Dinkelspiel, HE 2016 (22)	☆	☆	☆	--	--	☆	☆	☆	6
Erzen, M 2001 (23)	☆	☆	☆	--	--	--	☆	☆	5
Hermens, M 2021 (15)	☆	☆	☆	--	☆☆	☆	☆	☆	8
Ju, UC 2019 (17)	☆	☆	☆	--	☆☆	☆	☆	☆	8
Katagiri, A 2012 (24)	☆	☆	☆	--	--	☆	☆	☆	6
Kim, HS 2014 (13)	☆	☆	☆	--	☆☆	☆	☆	☆	8
Kumar, S 2011 (25)	☆	☆	☆	--	☆	☆	☆	☆	7
Lee, HY 2020 (26)	☆	☆	☆	--	☆☆	☆	☆	☆	8
Li, QW 2019 (16)	☆	☆	☆	--	☆	☆	☆	☆	7
Lu, J 2017 (27)	☆	☆	☆	--	☆☆	☆	☆	☆	8
Noli, S 2013 (28)	☆	☆	☆	--	☆	☆	☆	☆	7
Orezzoli, JP 2008 (29)	☆	☆	☆	--	☆	☆	☆	☆	7
Paik, ES 2018 (30)	☆	☆	☆	--	☆☆	☆	☆	☆	8
Park, JY 2018 (31)	☆	☆	☆	--	☆	☆	☆	☆	7
Ren, T 2017 (32)	☆	☆	☆	--	☆☆	☆	☆	☆	8
Scarfone, G 2014 (33)	☆	☆	☆	--	--	☆	☆	☆	6
Wang, S 2013 (34)	☆	☆	☆	--	☆☆	☆	☆	☆	8
Ye, S 2014 (35)	☆	☆	☆	--	☆☆	☆	☆	☆	8

### Meta-Analysis and Subgroup Analysis

**Figure 2:**
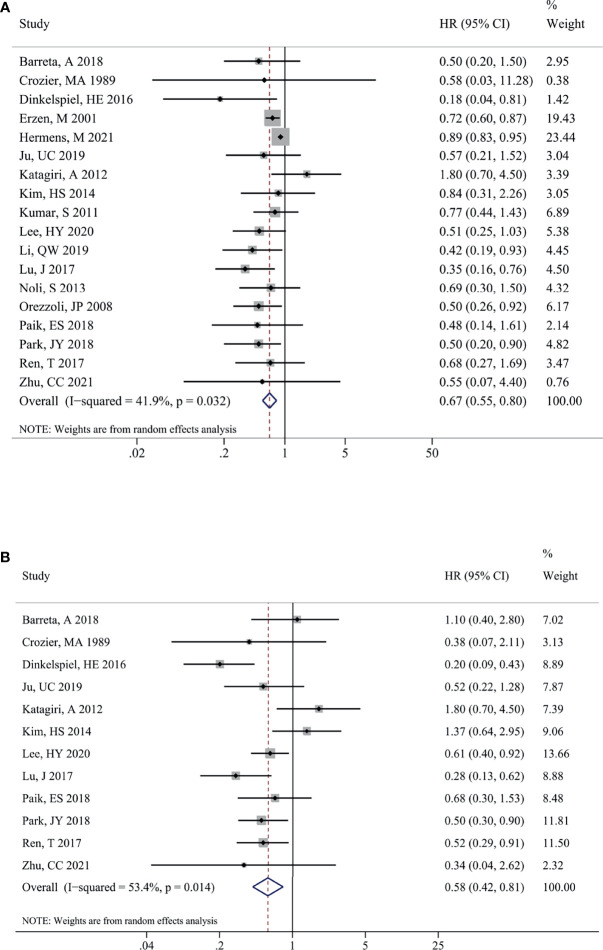
Impact of endometriosis on the prognosis of total OC. Forest plots showing the pooled results for endometriosis on overall survival **(A)** and progression-free survival **(B)** of total OC patients. OC, ovarian cancer.

The impact of endometriosis on the prognosis of total OC was presented in **Figure 2**. Eighteen studies reported the pooled results for OS of patients, and significant heterogeneity among studies were observed (I^2^ = 41.9%, P = 0.032). A random-effects model was utilized to pool the results. As shown in **Figure 2**, there was a significant difference in terms of OS within EAOC and non-EAOC groups [HR (95% CI) = 0.67 (0.55, 0.80), P < 0.001], suggesting that EAOC patients had better OS than non-EAOC patients. Similarly, EAOC patients had better PFS than non-EAOC patients [HR (95% CI) = 0.58 (0.42, 0.81), P = 0.001] in the random-effects model (I^2^ = 53.4%, *P*= 0.014) (**Figure 2**). Subgroup analysis indicated that no differences in OS were found between the OC patients with or without endometriosis [HR (95% CI) = 0.73 (0.38, 1.39), P = 0.332] in the univariate analysis subgroup. The results of other subgroups were consistent with the total pooled results. Subgroup analysis for PFS suggested that there was a statistically significant difference in Asian (P=0.005) and multivariate analysis subgroups (P=0.001), while there was no statistically significant difference in the America (P=0.169) and univariate analysis subgroups (P=0.260). In addition, whether confounding factors were adjusted or not was found to be one of the sources of significant heterogeneity for OS (**Table 3**).

**Table 3:** Outcomes of the subgroup analysis (total ovarian cancer patients).

Model	No. of studies	Heterogeneity test		Effect size	
		I^2^ (%)	P_H_	OR (95% CI)	*P* value
OS	18	41.9	0.032	0.67 (0.55, 0.80)	<0.001
Area					
Asia	10	0	0.443	0.57 (0.43, 0.76)	<0.001
America	5	0	0.486	0.56 (0.38, 0.82)	0.003
Europe	3	57.7	0.094	0.81 (0.68, 0.97)	0.023
Multivariate analysis					
Yes	13	38.1	0.080	0.62 (0.49, 0.79)	<0.001
No	5	43.3	0.133	0.73 (0.38, 1.39)	0.332
PFS	12	53.4	0.014	0.58 (0.42, 0.81)	0.001
Area					
Asia	9	44.5	0.071	0.63 (0.45, 0.87)	0.005
America	3	72.1	0.028	0.43 (0.13, 1.43)	0.169
Multivariate analysis					
Yes	8	31.9	0.173	0.60 (0.45, 0.80)	0.001
No	4	76.5	0.005	0.48 (0.14, 1.71)	0.260

**Figure 3:**
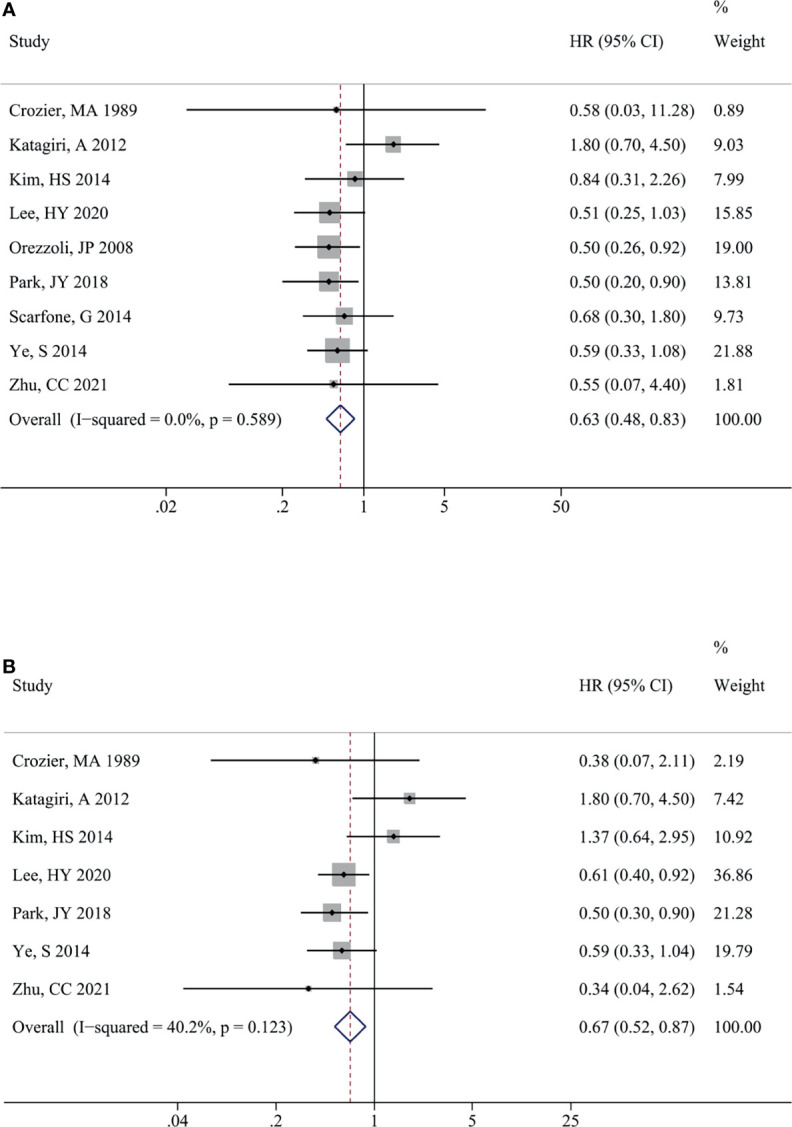
Impact of endometriosis on the prognosis of OCCC. Forest plots showed the pooled results for endometriosis on overall survival **(A)** and progression-free survival **(B)** of OCCC patients. OCCC, ovarian clear cell cancer.

The impact of endometriosis on OCCC prognosis is presented in **Figure 3**. There was no significant heterogeneity between the studies for OS (I^2^ = 0.0%, P = 0.589) and PFS (I^2^ = 40.2%, P = 0.123) therefore, fixed-effects models were used. Similarly, EAOC patients were associated with a better OS [HR (95% CI) = 0.63 (0.48, 0.83), P = 0.001] (**Figure 3**) and PFS [HR (95% CI) = 0.67 (0.52, 0.87), P = 0.002] (**Figure 3**) in comparison with non-EAOC patients. Subgroup analysis did not detect any significant difference in OS between the OCCC patients with or without endometriosis in the Europe [HR (95% CI) = 0.68 (0.28, 1.67), P =0.339] and univariate analysis subgroups [HR (95% CI) = 1.38 (0.61, 3.11), P =0.444]. The results of other subgroups were consistent with the total pooled results. Subgroup analysis for PFS suggested that there was statistical significance in the Asian (P=0.003) and multivariate analysis subgroups (P=0.001), while there was no statistical significance in the America (P=0.270) and univariate analysis subgroups (P=0.892) (**Table 4**).

**Table 4:** Outcomes of the subgroup analysis (OCCC patients).

Model	No. of studies	Heterogeneity test		Effect size	
		I^2^ (%)	P_H_	OR (95% CI)	*P* value
OS	9	0	0.589	0.63 (0.48, 0.83)	0.001
Area					
Asia	6	15.4	0.315	0.66 (0.47, 0.92)	0.015
America	2	0	0.924	0.50 (0.27, 0.94)	0.032
Europe	1	NA	NA	0.68 (0.28, 1.67)	0.399
Multivariate analysis					
Yes	6	0	0.951	0.57 (0.42, 0.76)	<0.001
No	3	0	0.497	1.38 (0.61, 3.11)	0.444
PFS	7	40.2	0.123	0.67 (0.52, 0.87)	0.002
Area					
Asia	6	47.9	0.087	0.68 (0.53, 0.88)	0.003
America	1	NA	NA	0.38 (0.07, 2.11)	0.270
Multivariate analysis					
Yes	4	36.2	0.195	0.64 (0.49, 0.83)	0.001
No	4	47.4	0.149	1.05 (0.49, 2.25)	0.892

### Publication Bias and Sensitivity Analysis

Egger’s test found no significant publication bias among the studies for PFS (P=0.945) of total OC patients, as well as the studies for OS (P=0.523) and PFS (P=0.728) of OCCC patients. There was significant publication bias among studies that reported the OS of total OC patients (P=0.002). The stability of the pooled results of the meta-analysis was evaluated using the trim-and-fill method, and the results found a significant difference in terms of OS within EAOC and non-EAOC groups, indicating stable results.

**Figure 4:**
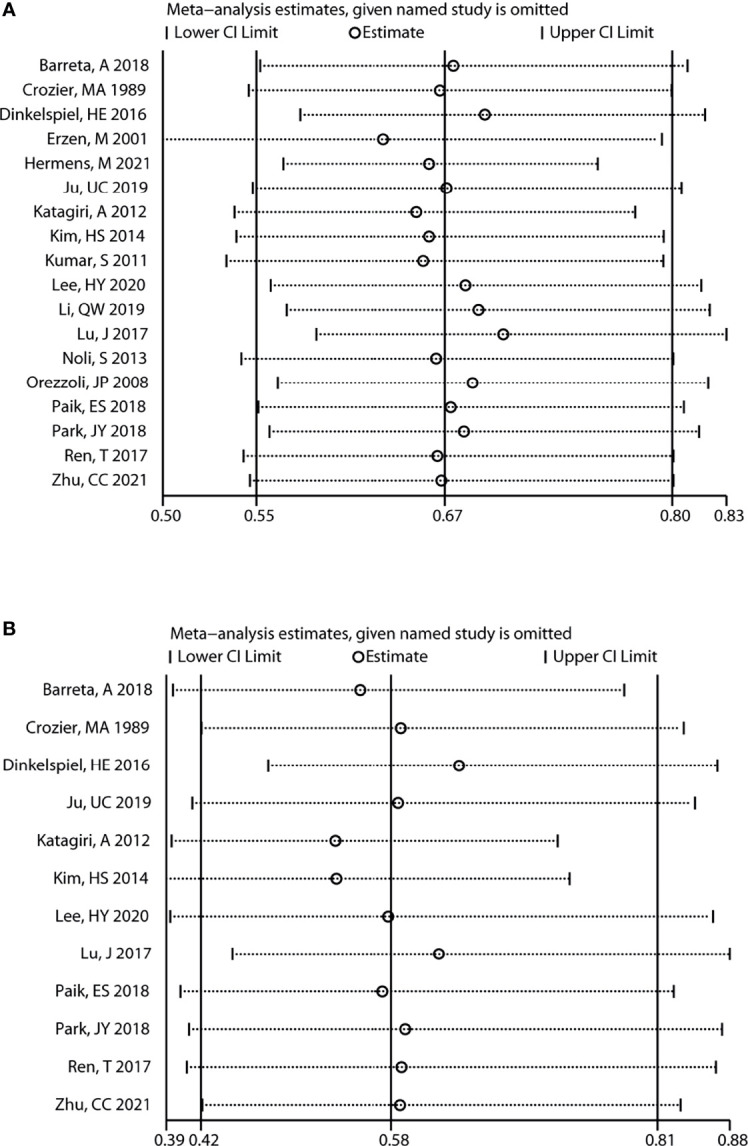
Sensitivity analysis for the results of total OC. Sensitivity analysis to evaluate the stability of the pooled results for the impact of endometriosis on overall survival **(A)** and progression-free survival **(B)** of total OC patients. OC, ovarian cancer.

**Figure 5:**
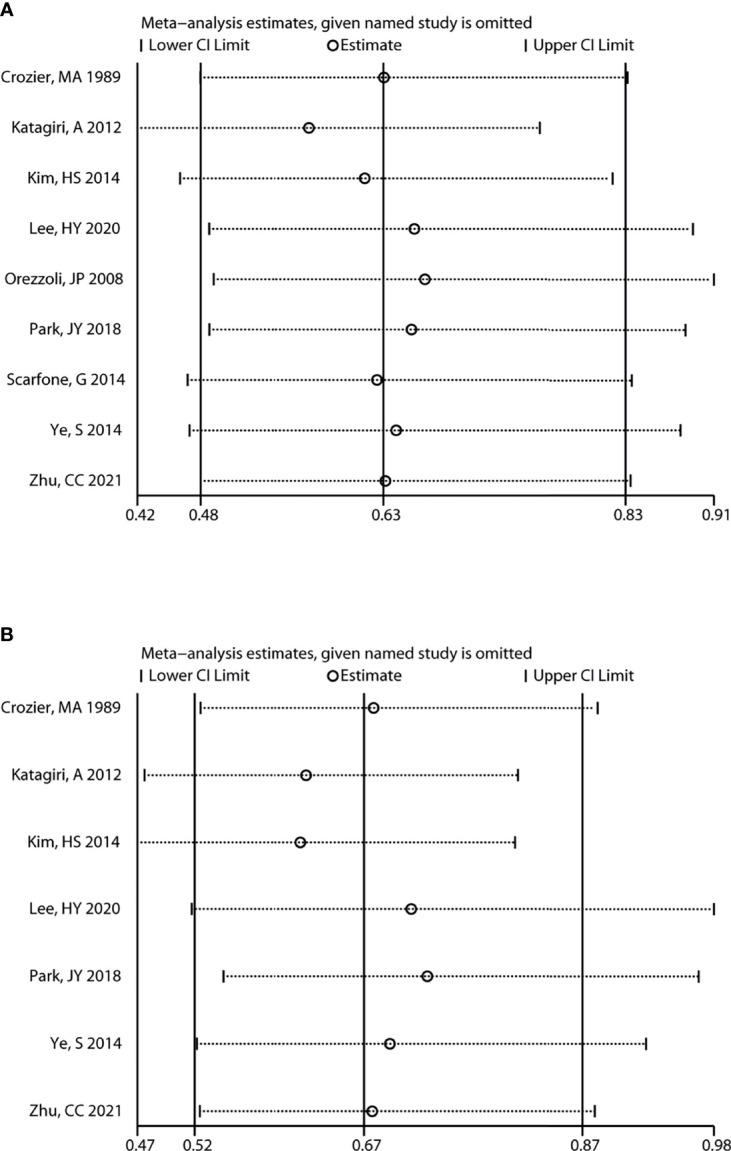
Sensitivity analysis for the results of OCCC. Sensitivity analysis to evaluate the stability of the pooled results for assessing the impact of endometriosis on the overall survival **(A)** and progression-free survival **(B)** of OCCC patients. OCCC, ovarian clear cell cancer.

In addition, sensitivity analysis revealed that the variation ranges of pooled results for OS [HR (95% CI) = 0.63 (0.50, 0.79) to 0.70 (0.59, 0.83), P < 0.05, **Figure 4**] and PFS [HR (95% CI) = 0.53 (0.39, 0.73) to 0.64 (0.47, 0.86), P < 0.05, **Figure 4**] of total OC patients were not significantly reversed. Similarly, the variation ranges of pooled results for OS [HR (95% CI) = 0.56 (0.42, 0.76) to 0.66 (0.49, 0.91), P < 0.05, **Figure 5**] and PFS [HR (95% CI) = 0.62 (0.47, 0.81) to 0.73 (0.55, 0.97), P < 0.05, **Figure 5**] of OCCC patients were not significantly reversed. These results suggest that the pooled results for all outcomes were stable and not significantly altered by any single study.

## Discussion

The prevalence of OC was 2.0-17.0% endometriosis patients, while the prevalence of endometriosis was 3.4-52.6% OC patients (37). A Nationwide Population-Based 14-year Cohort Study suggested that the prevalence of EOC in endometriosis patients ranged from 1.9 per 10000 in recalled endometriosis patients to 18.7 per 10000 in tissue-confirmed endometrioma (38). These data suggested that endometriosis was closely related to OC. EAOC is not a homogeneous group of malignancies and includes several histological subtypes. A previous study suggested that patients with endometriosis had specific histological subtypes of OC, including OCCC, EOC and low-grade serous OC, but not with mucinous or high-grade serous OC or borderline OC (9).

In this meta-analysis, we found that EAOC patients had a better OS in comparison to non-EAOC patients in both total OC and OCCC cohorts. Although there was significant publication bias among studies in terms of OS in the total OC cohort, the results of both the trim-and-fill method and the one-by-one elimination method suggested stability of the results of this meta-analysis. Consistent with our results, a higher OS rate in EAOC patients were also reported in the meta-analysis of Yang et al. (14) and Kim et al. (13). In addition, endometriosis-associated higher OS rates were also observed in the OCCC and mixed-subtype of OC subgroups in the meta-analysis of Yang et al. (14). However, another meta-analysis suggested that endometriosis had no influence on the OS of OCCC patients (12). Additionally, we also found that EAOC patients had better PFS than non-EAOC patients. However, no significant differences in terms of PFS within EAOC and non-EAOC patients were observed in previous meta-analyses (12–14). In these meta-analyses, less studies (4, 4 and 6 studies) in terms of PFS were included, while 12 studies were included in our study to explore the differences within EAOC and non-EAOC patients. More recent studies ranging from 2016 to 2021 were included. This might explain the reason for the different results in our meta-analysis and previous meta-analyses. For example, a recent study suggested that a significantly longer DFS was found in EAOC patients (51.9 months) than in non-EAOC patients (30.5 months) (39).

There was no significant difference in terms of PFS between EAOC and non-EAOC patients in the American subgroup in our study, suggesting that the study area might have influence on the results. Among the 21 included studies, the study of Hermens et al. (15) involved large amount of Dutch population-based cohort, and they suggested that patients with endometriosis had better OS than the patients without endometriosis. Similar results were observed in Asia and America populations in our study. Despite most of patients from the study of Hermens et al. (15), sensitivity analysis indicated that pooled results for all outcomes were stable and not significantly altered by their study. Additionally, we found that patients with endometriosis had better PFS than the patients without endometriosis in Asia population but not in America population. While PFS was not mentioned in the study of Hermens et al. (15).It was reported that OC patients with endometriosis were usually younger, and were usually diagnosed at an early stage (39). Significant differences between early stages (stages I and II, 73.8% vs. 41.8%, P < 0.00001) and low histological grades (grade I, 33.3% vs. 19.3%, P < 0.001) were found between EAOC and non-EAOC patients, suggesting a significant association of endometriosis with low tumor grades and early stage tumors (14). These might explain the better prognosis of EAOC patients, in that early-stage cancer might be a driver for better prognosis of EAOC patients rather than an association with endometriosis, as suggested in the study of Li et al. (16). However, Sharfrir et al. found a 29% decreased risk of death among OC patients who pre-diagnostic endometriosis, and association was stable after adjustment for FIGO stage, tumor histology, tumor characteristics and chemotherapy (40). Further studies should be performed to investigate the associations of endometriosis with prognosis of OC in age and stage matched studies. Women with endometriosis are more aware of physical discomfort, so they are more likely to see a doctor earlier, and may require more frequent gynecological ultrasound. This may explain the reason why OC patients with endometriosis were usually diagnosed at an early stage. On the other hand, the treatment of endometriosis involves continuous oral contraceptive use, which has been reported to reduce the risk of OC (41).

There were still some limitations in the current meta-analysis. (1) The adjuvant therapy regimens were not uniform among the included studies, which might affect the association between endometriosis and OS and PFS in OC patients. (2) The pooled results showed no statistical significance among studies that reported the crude HR, probably because that there was no significant difference in terms of OS or PFS of EAOC vs. non-EAOC in the univariate analysis of these studies and were therefore not included in the multivariate analysis. On the contrary, studies with statistical significance in univariate analysis and further implementation of multivariate analysis to correct for the influence of confounders on survival risk, endometriosis was more likely to be significantly associated with OS and PFS in OC patients. This might allow the pooled results of meta-analysis to overstate the strength of the association between endometriosis and risk of death. (3) All the included studies were retrospective studies; thus, recall and confounding biases were common and inevitable biases among them. Although multivariate analysis was performed in most studies to correct for the influence of confounding factors on the results, the inconsistency of correction factors might also affect the accuracy of the results.

In conclusion, this meta-analysis suggested that endometriosis was significantly associated with the OS and PFS of OC patients. EAOC patients tended to have longer OS and PFS than non-EAOC patients. Conducting higher quality prospective cohort studies with larger sample sizes are recommended to confirm the authenticity of the results.

## Data Availability Statement

The raw data supporting the conclusions of this article will be made available by the authors, without undue reservation.

## Author Contributions

PC acquired funding, collected data, conducted the statistical analysis, created tables and figures, and wrote the manuscript. C-YZ designed the study and helped in writing the manuscript. All authors contributed to the article and approved the submitted version.

## Funding

The present study was supported by The Natural Science Foundation of Liaoning Province (grant no. 2018010551–301) and the Shengjing Hospital of China Medical University (grant no. MF95). These funding supported in data analysis of the study and in writing the manuscript.

## Conflict of Interest

The authors declare that the research was conducted in the absence of any commercial or financial relationships that could be construed as a potential conflict of interest.

## Publisher’s Note

All claims expressed in this article are solely those of the authors and do not necessarily represent those of their affiliated organizations, or those of the publisher, the editors and the reviewers. Any product that may be evaluated in this article, or claim that may be made by its manufacturer, is not guaranteed or endorsed by the publisher.
