# Supplementary material for: Association Between Endometriosis and Prognosis of Ovarian Cancer: An Updated Meta-Analysis
Source: Front Oncol. 2022 Mar 31;12:732322. doi: 10.3389/fonc.2022.732322 (PMC9008736; doi:10.3389/fonc.2022.732322)
Supplement: Supplementary file 1 [file Table_1.docx]

The detailed retrieval strategy from PubMed database.

| Search | Query | Items found |
| --- | --- | --- |
| #1 | "ovarian neoplasms"[MeSH Terms] OR "ovarian neoplasms"[All Fields] OR "ovarian cancer"[All Fields] OR "ovarian carcinoma"[All Fields] OR "ovary carcinoma"[All Fields] OR "ovary cancer"[All Fields] OR "ovary neoplasm"[All Fields] | 106633 |
| #2 | "endometriosis"[MeSH Terms] OR "endometriosis"[All Fields] OR "endometrioses"[All Fields] | 29603 |
| #3 | "prognostic"[All Fields] OR "prognostica*"[All Fields] OR "prognostics"[All Fields] OR "prognosis"[MeSH Terms] OR "prognosis"[All Fields] OR "prognoses"[All Fields] OR "mortality"[MeSH Subheading] OR "mortality"[All Fields] OR "survival"[All Fields] OR "survival"[MeSH Terms] OR "survivability"[All Fields] OR "survivable"[All Fields] OR "survivals"[All Fields] OR "survive"[All Fields] OR "survived"[All Fields] OR "survives"[All Fields] OR "surviving"[All Fields] | 3623983 |
| #4 | #1 AND #2 AND #3 | 702 |

The detailed retrieval strategy from Embase database.

| Search | Query | Items found |
| --- | --- | --- |
| #1 | ('ovarian carcinoma'/exp OR 'ovarian carcinoma' OR 'ovarian neoplasms'/exp OR 'ovarian neoplasms' OR 'ovarian cancer'/exp OR 'ovarian cancer' OR 'ovary carcinoma'/exp OR 'ovary carcinoma' OR 'ovary cancer'/exp OR 'ovary cancer' OR 'ovary neoplasm'/exp OR 'ovary neoplasm') | 147853 |
| #2 | ('endometriosis'/exp OR endometriosis OR endometrioses) | 38137 |
| #3 | (prognostic OR 'prognosis'/exp OR prognosis OR 'survival'/exp OR survival OR 'mortality'/exp OR mortality OR prognostica*) | 3221766 |
| #4 | #1 AND #2 AND #3 | 713 |

The detailed retrieval strategy from Web of Science database

| Search | Query | Items found |
| --- | --- | --- |
| #1 | ALL FIELDS: ((ovarian carcinoma) OR (ovarian neoplasms) OR (ovarian cancer) OR (ovary carcinoma) OR (ovary cancer) OR (ovary neoplasm)) | 144303 |
| #2 | ALL FIELDS: (Endometriosis OR endometrioses) | 24133 |
| #3 | ALL FIELDS: (prognostic OR prognosis OR survival OR mortality OR prognostica*) | 2430426 |
| #4 | #1 AND #2 AND #3 | 516 |
